# Mutational analysis and clinical investigations of medically diagnosed GSD 1a patients from Pakistan

**DOI:** 10.1371/journal.pone.0288965

**Published:** 2023-11-30

**Authors:** Bushra Gul, Sabika Firasat, Tayyaba Shan, Raeesa Tehreem, Kiran Afshan

**Affiliations:** 1 Department of Zoology, Faculty of Biological Sciences, Quaid-i-Azam University, Islamabad, Pakistan; 2 Department of Biosciences, University of Wah, Wah Cantt, Pakistan; Sohag University Faculty of Medicine, EGYPT

## Abstract

Glycogen storage disease type I (GSD I) is a rare autosomal recessive inborn error of carbohydrate metabolism caused by the defects of glucose-6-phosphatase complex (*G6PC*). Disease causing variants in the *G6PC* gene, located on chromosome 17q21 result in glycogen storage disease type Ia (GSD Ia). Age of onset of GSD Ia ranges from 0.5 to 25 years with presenting features including hemorrhage, hepatic, physical and blood related abnormalities. The overall goal of proposed study was clinical and genetic characterization of GSD Ia cases from Pakistani population. This study included forty GSD Ia cases presenting with heterogeneous clinical profile including hypoglycemia, hepatomegaly, lactic acidosis i.e., pH less than 7.2, hyperuricemia, seizures, epistaxis, hypertriglyceridemia (more than180 mg/dl) and sometimes short stature. All coding exons and intron-exon boundaries of *G6PC* gene were screened to identify pathogenic variant in 20 patients based on availability of DNA samples and willingness to participate in molecular analysis. Pathogenic variant analysis was done using PCR-Sanger sequencing method and pathogenic effect predictions for identified variants were carried out using PROVEAN, MutationTaster, Polyphen 2, HOPE, Varsome, CADD, DANN, SIFT and HSF software. Overall, 21 variants were detected including 8 novel disease causing variants i.e., G6PC (NM_000151.4):c.71A>C (p.Gln24Pro), c.109G>C(p.Ala37Pro), c.133G>C(p.Val45Leu), c.49_50insT c.205G>A(p.Asp69Asn), c.244C>A(p.Gln82Lys) c.322A>C(p.Thr108Pro) and c.322A>C(p.Cys284Tyr) in the screened regions of *G6PC* gene. Out of 13 identified polymorphisms, 3 were identified in heterozygous condition while 10 were found in homozygous condition. This study revealed clinical presentation of GSD Ia cases from Pakistan and identification of novel disease-causing sequence variants in coding region and intron-exon boundaries of *G6PC* gene.

## Introduction

Defects in enzymatic pathways of liver are associated with altered metabolism leading to hypoglycemia ± hepatomegaly and/or liver disease in hepatic forms of glycogen storage disorder (GSD) [1]. Based on affected enzyme and its relative expression in the liver, kidney, skeletal muscle, or heart, the clinical manifestations of GSDs vary from one disorder to the other [2]. Type I glycogen storage disease (GSD I) is the commonest most autosomal recessive form that typically presents in early infancy [3]. The catalytic subunit of microsomal glucose-6-phosphatase (G-6-Pase; E.C. 3.1.3.9) plays a pivotal role in glycogenolysis and gluconeogenesis catalyzing the last step of both metabolic pathways. Its deficiency leads to glycogen storage disease type Ia (GSD Ia; Von Gierke Disease; MIM #232200), which is usually characterized by hepatomegaly, hypoglycemia, lactic acidemia, hyperuricemia, hyperlipidemia. Untreated patients may have a cushingoid appearance, failure to thrive, an enlarged liver, protuberant abdomen, and delayed motor development [4, 5]. Cerebral damage resulting from recurrent hypoglycemic episodes may lead to abnormal cognitive development [6]. The demonstration of a reduced G-6-Pase activity measured in a fresh liver biopsy specimen is still considered the gold standard for verification of the clinical diagnosis. However, in 1993 the gene (*G6PC*; GDB 231927) spanning 12.5 kb on chromosome 17 and consisting of 5 exons coding for the enzyme was cloned [7]. The protein encoded by this gene contains 357 amino acids and is an endoplasmic reticulum (ER) membrane associated protein containing the ER retention signal, and possesses six putative membrane spanning segments [8]. To date 146 sequence variations have been identified in *G6PC* gene related to GSD 1a, which have been documented from various countries (The Human Gene Mutation Database (HGMD1). Available at: http://www.hgmd.cf.ac.uk/ac/index.php, Accessed: 20July 2022).

Owing to recessive inheritance pattern of GSD Ia, its incidence is higher in populations with customary consanguineous marriages like Pakistan, necessitating comprehensive clinical and genetic studies on this disease from our local population. However there are only few reports from Pakistan focusing on the biochemical findings and clinical manifestations of the disease in children with GSD 1a, and a report on the disease-causing variation identified in a Pakistani case, described that disease causing variants may not be comprehensive, and there may be additional mutations yet to be identified for Pakistani patients with GSD Ia [9, 10]. To understand molecular basis of GSD Ia in Pakistani population for potential therapeutic targets there is a need of extensive research regarding contributing genetic and environmental risk factors of GSD Ia. Therefore, the present study was aimed to check clinical heterogeneity among forty Pakistani GSD Ia patients presented at two tertiary care hospitals. We aimed to investigate the variants in *G6PC* gene among 20 cases based on willingness to participate in molecular analysis, to identify the underlying molecular defects leading to GSD1a phenotype.

## Methods

The study was carried out at Molecular Biology Lab, Quaid-i-Azam University (QAU), Islamabad, Pakistan after approval from Bioethical Committee of Faculty of Biological Sciences, QAU. Patients affected with GSD-1a, diagnosed at Neurology and Gastroenterology departments of Children Hospital Lahore (CHL) and Pakistan Institute of Medical Sciences (PIMS), Islamabad were recruited in this prospective study. Blood samples of patients and available unaffected family members were collected along with the family history and clinical data after informed written consent. Forty patients (35 with parental cousin marriages) were diagnosed clinically for enlarged liver and kidneys, growth retardation and short stature, abnormal levels of glucose, lactate, uric acid, triglycerides, and cholesterol. Frequencies of observed clinical features and mean values of diagnostic tests were calculated using SPSS 21.0. Blood samples were stored in EDTA containing tubes and DNA extraction was performed using phenol chloroform method [11]. Primers of all 5 exons of *G6PC* gene (ENST00000253801, NM_000151) were designed by using Primer3 software (http://bioinfo.ut.ee/primer3-0.4.0/) (Table 1). The PCR reactions were carried out using protocol described by Gul *et al*., 2022 [12]. The amplified PCR products were loaded on the 1.5% agarose gel along with 1 kb size ladder to evaluate product size. The purification was done by using DNA purification Kit (Wiz Bio Solutions, Seongnam, Korea) and purified products were sent for commercial Sanger’s sequencing. The sequenced data was analyzed by using Sequencher 5.4.6 software. Pathogenicity prediction for each variant was done by various bioinformatics tools named Mutalyser (https://mutalyzer.nl/), MutationTaster (http://www.mutationtaster.org/), PROVEAN (http://provean.jcvi.org/index.php), Polyphen-2 (http://genetics.bwh.harvard.edu/pph2/), Mutation assessor (http://mutationassessor.org/r3/), SIFT (http://sift.jcvi.org/), HOPE (https://www3.cmbi.umcn.nl/hope/method), Varsome (https://varsome.com/), CADD (https://cadd.gs.washington.edu/snv), HSF (Human Splice Site Finder) software version 3.0 (www.umd.be/HSF3/) to determine effects of sequence variations on exonic splicing signals. American College of Medical Genetics and Genomics (ACMG) classification is used for variant classification.

**Table 1:** Primers used for amplification of exons 1, 2, 3, 4 and 5 of *G6PC* gene.

Exon No.	Primer Sequence (5’ ➝ 3’)		Product Size (bp)
**1**	F	TTGAGTCCAAAGATCAGGGC	483 bp
	R	TGAATAGCCTGGGGAAAGCA	
**2**	F	CCACCCAGTTCTCCCTTCTA	519 bp
	R	CTTTCTCAGGACACAGCGCT	
**3**	F	GGTAGATGGGTGGATAGGGG	289 bp
	R	AGAATACGTGGTGTGTCAGC	
**4**	F	AAAATTCCACTGAGAGCACCT	358 bp
	R	ACCCACAGAAATGCTAACAGT	
**5a**	F	GCAGAACGGATGGCATGTCA	385 bp
	R	AGCTCTCCCTGTACATGCTG	
**5b**	F	GTGGACTCTGGAGAAAGCCC	524 bp
	R	GACCCTCCAATCTGCCATCC	

## Results

A total of forty patients diagnosed with GSD Ia, included in this study showed diverse clinical symptoms (see S1 Table). Frequency of disease was observed more in males as out of 40 enrolled cases 26 (65%) were males and 14 (35%) patients were females. Parental consanguinity was observed in 35 (87.5%) cases with 23 (57.5%) showing family history of disease. Out of forty enrolled cases, 23 (57.5%) patients died within 6 months because of disease severity. Hepatomegaly was observed in all cases (100%), however hepatic adenomas were present in 2 (5%) cases. 10 (25%) cases had a history of seizures. Epistaxis was observed in 7 (17.5%) cases. Delayed motor development was observed in 5 (12.5%) however 3 (7.5%) showed cushingoid appearance. Osteopenia was seen in 9 (22.5%) cases and 15 (37.5%) had inflammatory bowel disease (see S1 Table). The mean values of diagnostic tests performed for GSD Ia cases are shown in Table 2.

**Table 2:** Age and the mean values of diagnostic tests performed for GSD Ia cases included in this study.

Continuous variables	Mean± Std. Deviation
Age	7.913±4.9625
Hypoglycemia (mg/dL)	58.500±6.5984
High microalbuminuria	166.725±105.1239
Hyperuricemia	6.215±.9989
Proteinuria	192.225±53.1994
Hypertriglyceridemia	279.075±59.9912
Lactic Acidosis	5.728±.8249

Std = Standard

For molecular analysis blood samples of 20 cases were collected based on patient’s willingness to participate in genetic analysis as well as blood transfusion records. Upon sequencing of coding exons, their flanking intronic regions and 3’ as well as 5’ untranscribed regions (UTRs) of *G6PC* (chromosome:GRCh38:17:42900197:42913969:1) gene in 20 patients, overall, 21 variants were detected (Table 3). Out of 21 identified variants there were 8 novel disease-causing variants (5 homozygous and 3 heterozygous) as predicted by mutation taster in the coding regions of exon 1, 2 and 5b of *G6PC* gene and 13 polymorphisms (10 homozygous and 3 heterozygous). The identified disease-causing variants were neither found in 1000G nor in Exome Aggregation Consortium” ExAC–composed of 60,706 unrelated individuals, and the Online Archive of Brazilian Mutations.

**Table 3:** List of identified variants in *G6PC* gene in GSD 1a cases identified in this study.

HGVS	Patient ID	Exon	Zygosity	Polyphen 2 Prediction (Score)	ACMG Classification	Functional domain	SIFT (P/S)	Novelity/ID
G6PC(NM_00151.4):c.49_50insT	2	E1	Homo	NA	Likely pathogenic	5’UTR	N/A	N
G6PC(NM_00151.4):c.71A>C(p.Gln24Pro)	11	E1	Hetero	B (0)	Likely benign	CDS	T 0.32	N
G6PC(NM_00151.4):c.109G>C(p.Ala37Pro)	11	E1	Homo	PD (0)	Uncertain significance	CDS	APF 0.05	N
G6PC(NM_00151.4):c.133G>C(p.Val145Leu)	5	E1	Hetero	B (0.079)	Uncertain significance	CDS	T 0.53	N
G6PC(NM_00151.4):c.205G>A(p.Asp69Asn)	5	E1	Homo	PD (1)	Uncertain significance	CDS	APF 0.02	N
G6PC(NM_00151.4):c.244C>A(p.Gln82Lys)	9	E2	Homo	PD (0.907)	Uncertain significance	CDS	T 0.21	N
G6PC(NM_00151.4):c.322A>C(p.Thr108Pro)	4	E2	Homo	PD (1)	Likely pathogenic	CDS	D 0.02	N
G6PC(NM_00151.4):c.322A>C(p.Cys284Tyr)	19	E5	Hetero	PD (0.771)	Uncertain significance	CDS	T 0.08	N
NC_000017.9:g.38301348_38301349insT	2	E1	Homo	NA	Likely benign	5’UTR	N/A	N
NC_000017.9:g.38304430delA	10,18	E2	Homo	NA	Uncertain significance	I	N/A	N
NC_000017.9:g.38304606delG>T	7,20	E2	Hetero	NA	Benign	I	N/A	N
NC_000017.9:g.38304772T>C	1,2,4,18,20	E2	Homo	NA	Benign	I	N/A	rs2593595
NC_000017.9:g.38308244_38308245insA	9	E3	Homo	NA	Benign	I	N/A	N
NC_000017.9:g.38308101T>A	3	E3	Homo	NA	Benign	CDS	N/A	N
NC_000017.9:g.38315015T>C	9,3,2	E4	Hetero	NA	Benign	I	N/A	rs161622
NC_000017.9:g.38309808_38309809insA	2,8	E4	Homo	NA	Uncertain significance	I	N/A	N
NC_000017.9:g.38309815_38309816insC	6	E4	Homo	NA	Uncertain significance	I	N/A	N
NC_000017.9:g.38309809T>G	2	E4	Homo	NA	Benign	I	N/A	N
NC_000017.9:g.38316992T>C	7,10,18,20	E5	Hetero	NA	Likely benign	3’UTR	N/A	rs2229611
NC_000017.9:g.38311697G>A	9	E5	Homo	NA	Benign	CDS	N/A	N
NC_000017.9:g.38312116A>C	1	E5	Homo	NA	Benign	3’UTR	N/A	N

PD: Probably damaging, B: Benign, HGVS: Human Genome Variation Society, N/A: Not applicable, CDS: Coding sequence, I: Intron, N: Novel, 3’UTR: 3’ Untranslated region. APF: Affected protein function, D: Damaging, T: Tolerant.

**Fig 1:**
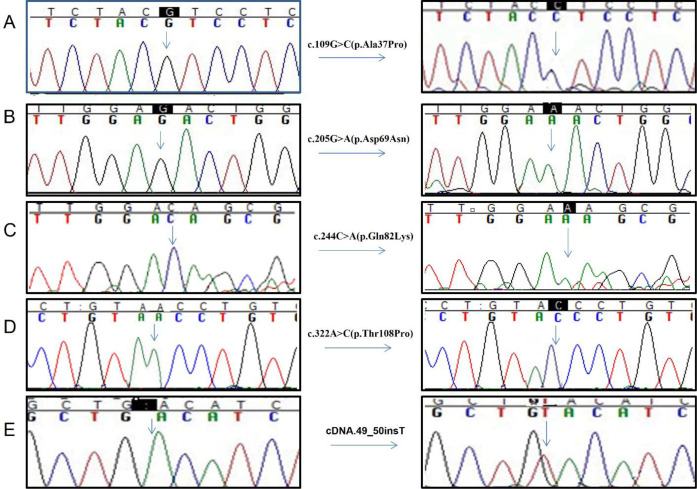
Electropherograms of homozygous disease-causing variants identified in *G6PC* gene. **A**. Electropherogram showing c.109G>C (p.Ala37Pro) in patient 11 in exon 1 of *G6PC* gene. **B**. Electropherogram showing c.205G>A (p.Asp69Asn) in patient ID 5 in exon 1 of *G6PC* gene. **C**. Electropherogram showing c.244C>A (p.Gln82Lys) in patient ID 9 in exon 2 of *G6PC* gene. **D**. Electropherogram showing c.322A>C (p.Thr108Pro) in patient ID 4 in exon 2 of *G6PC* gene. **E**. Electropherogram showing c.49_50insT identified in patient ID 2 in exon 1 of *G6PC* gene.

**Fig 2:**
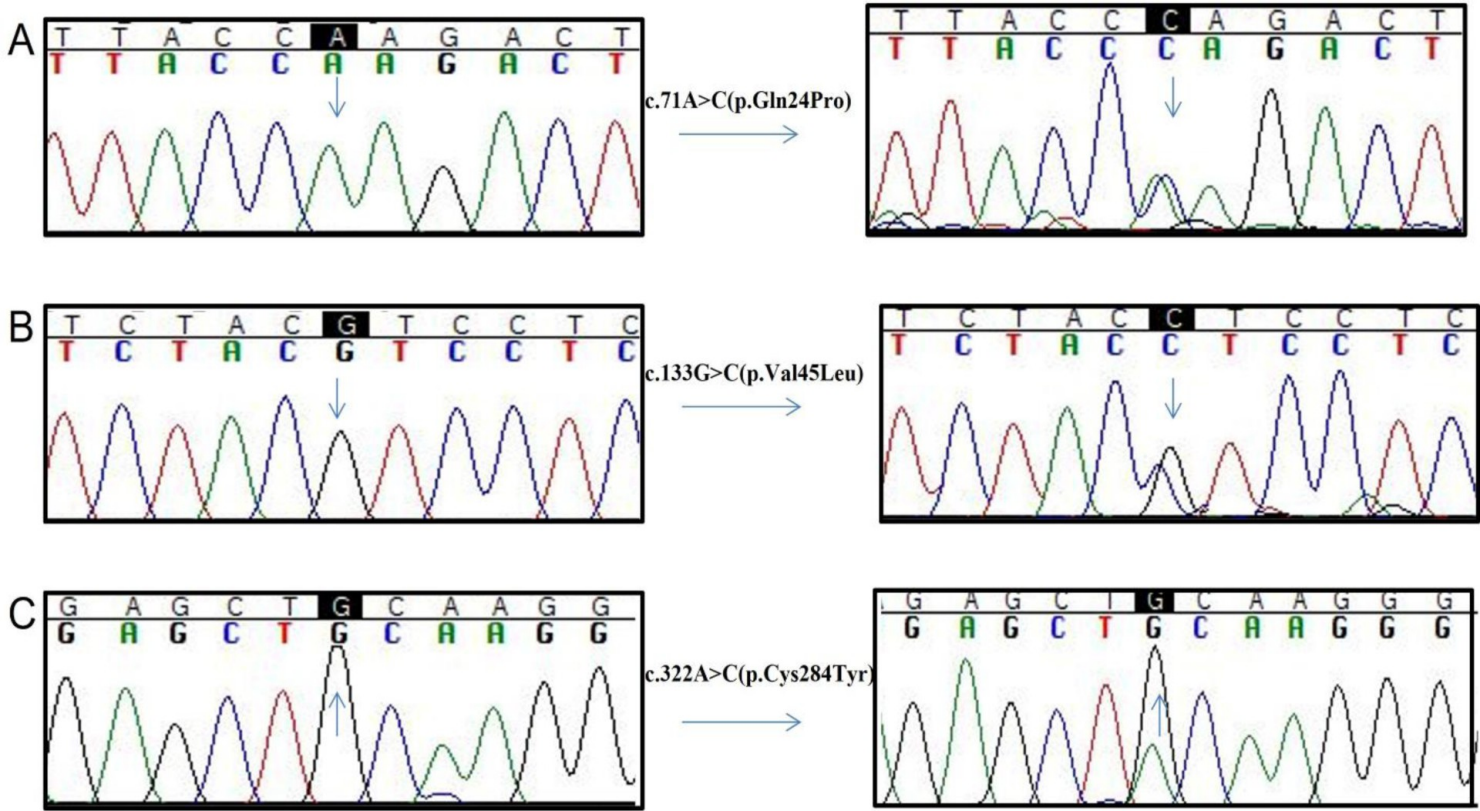
Electropherograms showing heterozygous disease-causing variants identified in *G6PC* gene. **A**. Electropherogram showing c.71A>C (p.Gln24Pro)identified in exon 1 of *G6PC* gene. **B**. Electropherogram showing c.133G>C (p.Val45Leu) identified in exon 1 of *G6PC* gene. **C**. Electropherogram showing c.322A>C (p.Cys284Tyr) identified in exon 5b of *G6PC* gene.

Five novel homozygous disease causing variants include G6PC (NM_000151.4):c.109G>C (p.Ala37Pro (patient ID 11), c.205G>A (p.Asp69Asn) (patient ID 5), c.49_50insT (patient ID 2) in exon 1 of *G6PC* gene; c.244C>A (p.Gln82Lys) (patient ID 9) and c.322A>C(p.Thr108Pro) (patient ID 4) in exon 2 of *G6PC* gene (Fig 1A–1E). Three novel identified heterozygous disease causing variants include c.71A>C (p.Gln24Pro) (patient ID 11), c.133G>C (p.Val45Leu) (patient ID 5) in exon 1 of *G6PC* gene and c.322A>C (p.Cys284Tyr) (patient ID 19) in exon 5 of *G6PC* gene (Fig 2A–2C) (Table 3). All variants predicted to be disease causing by mutation taster were predicted to be damaging by Polyphen2 (Table 3). These variants were not found in ExAC. Hope analysis showed that the original wild-type residue and newly introduced mutant residue differ in properties for each novel missense variant playing role in disease pathogenicity.

**Fig 3:**
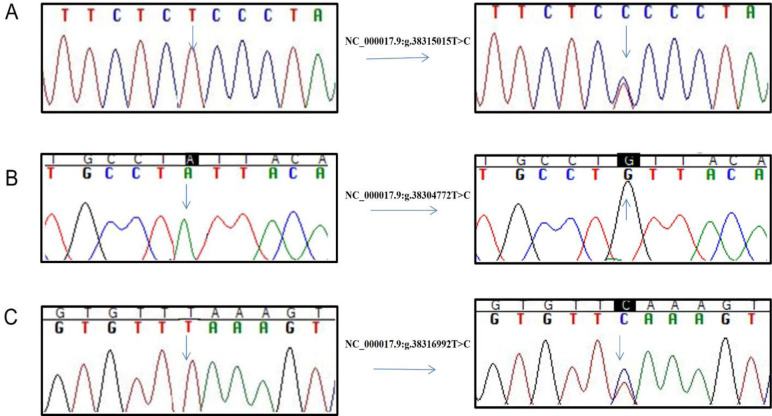
Electropherograms showing reported polymorphisms in *G6PC* gene identified in this study. A- g.38315015T>C identified in heterozygous state (rs161622), B. g.38304772T>C identified in homozygous state (rs2593595) C. g.38316992T>C identified in heterozygous state (rs2229611).

**Fig 4:**
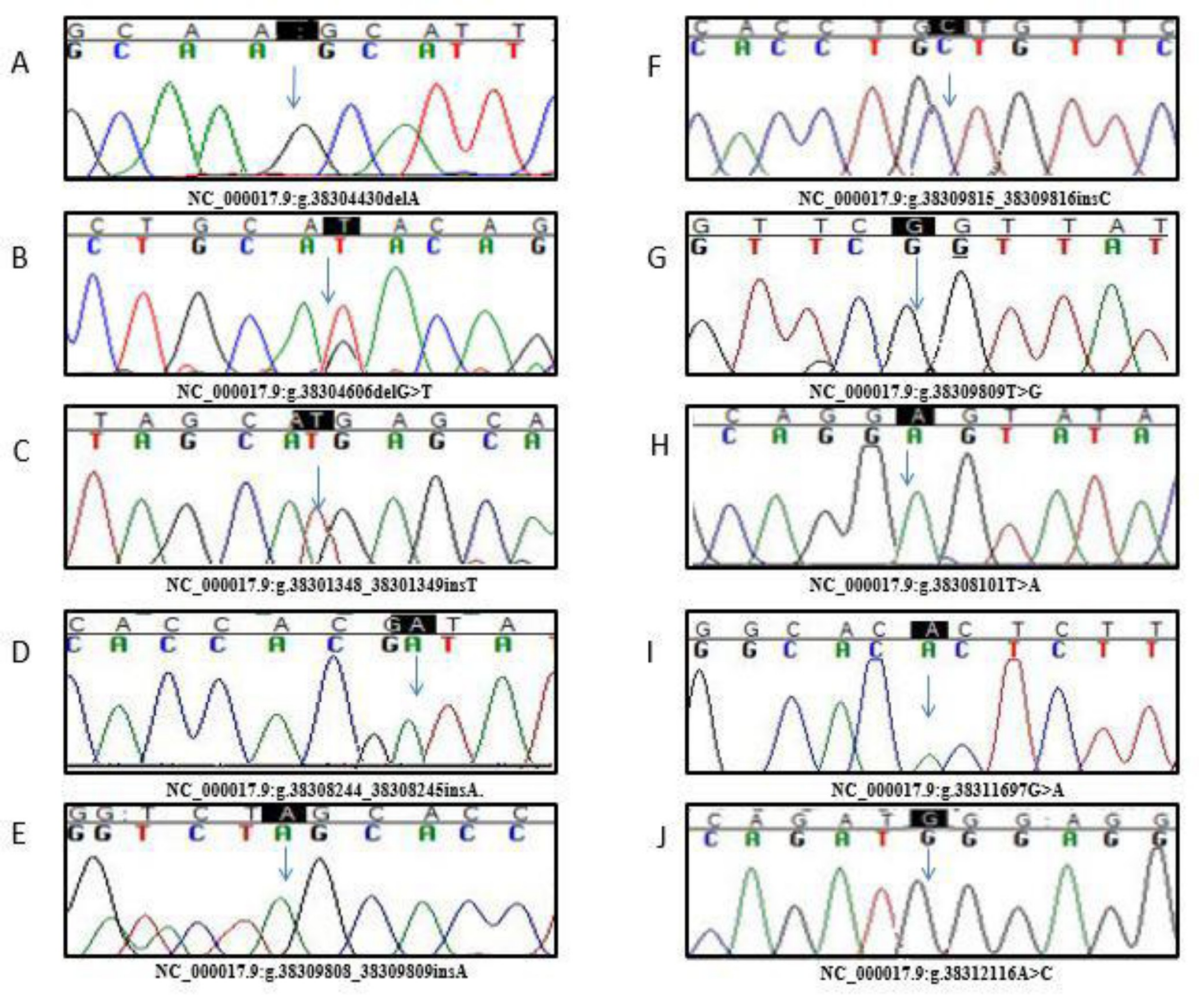
Electropherograms showing novel polymorphisms identified in *G6PC* gene in this study. **A**. g. g.38304430delA (homozygous). **B**. g.38304606delG>T (heterozygous), **C**. g.38301348_38301349insT (homozygous). **D**. g.38308244_38308245insA (homozygous). **E**. g.38309808_38309809insA (homozygous) **F**. g.38309815_38309816insC (homozygous), **G.** g.38309809T>G g.8481T>G (homozygous). **H**. g.38308101T>A (homozygous). **I** g.38311697G>A (homozygous) **J**. g.38312116A>C (homozygous).

Among three known polymorphisms identified in this study, a homozygous variant g.38304772T>C (rs2593595) was identified in 25% cases and two heterozygous variants i.e., g.38315015T>C (rs161622) and g.38316992T>C (rs2229611) were identified in 15% and 20% of cases respectively (Fig 3A–3C, Table 3). The novel polymorphisms identified in GSD Ia cases include g.38304430delA, g.38308244_38308245insA, g.38309808_38309809insA, g.38309815_38309816insC, g.38308101T>A, g.38311697G>A, g.38312116A>C and g.38309809T>G in homozygous states whereas variant i.e., g.38304606delG>T was found in heterozygous state (Fig 4A–4J, Table 3).

## Discussion

Glucose 6-phosphatase (*G6PC*) enzyme catalyzes the hydrolysis of glucose-6-phosphate (G6P) to produce inorganic phosphate and glucose in liver and kidney cells. The glucose produced is then transported out of the cell to contribute in maintenance of the blood glucose level even during starvation [13]. Disease causing variants in the *G6PC* gene result in defective glucose-6-phosphatase activity causing storage of glycogen in liver and kidney cells leading to glycogen storage disease type Ia (GSD Ia). Although there have been some studies on the incidence of GSD Ia in Pakistan, there is still a need for more comprehensive epidemiological data of the disease. This would include data on the incidence, prevalence, and distribution of the disease across different regions and populations in Pakistan. GSD Ia is a rare but serious metabolic condition that runs in families as an autosomal recessive disorder [8, 14]. There have been some studies on the genetic basis of GSD Ia from Pakistan, there is still much to be learned about the specific mutations and genetic variants that are most commonly associated with the disease in this population. This knowledge could help to design more effective diagnostic and treatment approaches. Buildup of glycogen in the liver and kidneys, cause progressive hepatomegaly and nephromegaly. Hypercholesterolemia, hypertriglyceridemia, hyperuricemia, and lactic acidemia are all metabolic implications of elevated cytoplasmic G6P levels [15]. Analysis of *G6PC* pathogenic variants is required to acquire differential clinical diagnosis [16, 17]. To date, various pathogenic variants have been reported in the *G6PC* gene worldwide, including missense (the most prevalent form), nonsense, insertion/deletion and splice site variants, but there is no significant data from Pakistan [14]. Characterization of *G6PC* and identification of disease-causing variants in this gene provide a DNA-based tool to diagnose patients clinically suspected for GSD Ia. Moreover, disease causing variant analysis of a family at risk of conceiving offspring with GSD Ia offers genetic counseling. Furthermore for affected individuals, early diagnosis allows the employment of adequate metabolic control strategies and treatments to prevent complications and thus increases the quality of life [18]. Autosomal recessive disorders are prevalent in Pakistan because of the high rate of consanguineous marriages [19]. Incidence of hepatic glycogenesis is unknown for Pakistan, and there have been no in-depth disease causing variant investigations of GSDs to date.

Current study focuses on clinical and genetic analysis of GSD 1a cases from Pakistan. Clinical analysis was performed on 40 GSD 1a cases, with presenting symptoms of seizures, irritability and increased respiratory rate caused by hypoglycemia and hyperlactacidaemia as well as hepatomegaly was predominantly present (see S1 Table).

The study identified that males (65%) were more affected as compared to females (35%). In current study the ratio of males identified with disease was more than the females. The disease being autosomal, is not linked with any of the gender. So, it could be due to underdiagnosis. 70% of the patients belonged to the age group 0–10 years in which the disease appeared during early ages. 87.5% of cases in this study belonged to consanguineous families. 57.5% patients could not survive disease severity due to lack of proper disease management resulting in complications including severity of hypoglycemic events leading to high mortality rate as reported previously by Ai et al., 2020 [20]. Seizures and delayed motor development were observed in 25% and 12.5% cases respectively, as observed in previous studies [21, 22]. Three patients were observed with cushingoid appearance and osteopenia was found in 9 cases (see S1 Table). Cushingoid appearance, delayed motor development and osteopenia is attributed to untreated GSD Ia [23, 24]. These complications are attributed to hypothalamic-pituitary-adrenal (HPA) axis stimulation due to chronic hypoglycemic stress causing elevated glucocorticoid secretion [25]. In our study cohort, inflammatory bowel disease (IBD) was present in 15 patients (37.5%) (see S1 Table), that is higher as studies have reported an occasional presence of IBD in GSD Ia cases [26, 27]. Disease causing variant analysis of GSD Ia in 20 selected cases identified four novel missense variants i.e., c.109G>C(p.Ala37Pro), c.205G>A(p.Asp69Asn), c.244C>A(p.Gln82Lys) and c.322A>C(p.Thr108Pro) and an insertion c.49_50insT in homozygous condition. All these variants were predicted to be disease causing according to Polyphen 2 and SIFT prediction. In addition, we also identified three missense variants including c.71A>C (p.Gln24Pro), c.133G>C(p.Val45Leu) and c.322A>C(p.Cys284Tyr)in heterozygous conditions in GSD Ia affected cases. All these disease-causing variants have not been reported yet. Variants identified in coding sequence of exon 1 are c.71A>C (g.151A>C) with protein change p.Gln24Pro, c.109G>C (g.189G>C) with protein change p.Ala37Pro, c.133G>C (g.213G>C) and c.205G>A (g.285G>A) with amino acid changes p.Val45Leu and p.Asp69Asn respectively. An insertion disease causing variant also identified in 5’UTRc.49_50insT. The variant c.322A>C (g.3226A>C) and c.244C>A (g.3148C>A) identified in coding sequence of exon 2 are associated with protein changes p.Thr108Pro (replacement of a hydrophilic with hydrophobic amino acid) and p.Gln82Lys (replacement of acidic with basic amino acid) respectively and were predicted to be damaging with Polyphen score of 1 and 0.907. A missense damaging variant c.322A>C (g.10407G>A) with amino acid change i.e., p.Cys284Tyr (replacement of sulfur containing amino acid with aromatic amino acid) is identified in coding sequence of exon 5 which is predicted to be damaging with Polyphen score of 0.771 and SIFT score of 0.08. Sequencing of exon 1 of patient ID 5 and patient ID 11 identified homozygous i.e. c.205G>A (p.Asp69Asn), c.109G>C(p.Ala37Pro) and heterozygous i.e., c.133G>C(p.Val45Leu), c.71A>C(p.Gln24Pro) disease causing variants in both patients, however sequencing identified no polymorphism in these two cases. Identified homozygous variants in both patients were predicted to be probably damaging according to Polyphen 2 prediction, while both heterozygous variants were benign highlighting the need to sequence remaining non coding and regulatory sequences of gene to identify the molecular genetic defects underlying the disease phenotype. For validation of all variants, sequencing with both forward and reverse primers was done. The previously reported hotspot pathogenic variant i.e., p.Arg83Cys that was detected in 50% of alleles in French and Tunisian patients, 80% of Sicilian and 100% of alleles in Ashkenazi Jewish patients is not identified in this study cohort [28]. One of the possible explanation is small sample size of present study and a need of future large cohort studies that might identify such previously known hotspot variants from our Pakistani population.

Glucose 6 phosphatase is anchored in ER membrane by nine transmembrane helix structures, the amino terminus lies in the lumen of ER while carboxy terminal in cellular cytoplasm [29] and all of our identified missense disease-causing variants are detected in the transmembrane helix structures of *G6PC*. Shieh and Angaroni., 2003 have suggested that the majority of helical missense variants cause decreased stability of *G6PC* protein compared to the wild-type enzyme [30]. Although the functional studies could not be performed to confirm damages caused by pathogenic variants at protein level but the bioinformatic analysis demonstrated the pathogenic statuses of identified variants. Hence identification of homozygous missense disease-causing variants in five cases and heterozygous variants in three cases provide the molecular genetic basis of clinical manifestations of the GSD Ia in these patients.

In fourteen cases showing clinical symptoms of GSD Ia, no disease-causing variants in coding regions was found, which highlights the ratio of *G6PC* disease causing variants in our study to be 30%, however there is still need of further molecular studies since due to overlapping clinical presentations of glycogen storage disease types. Identification of variants in this study provided additional tool for genetic counselling.

Despite many advances at molecular genetics level, there are still a number of inconsistencies in GSD 1a that remained unresolved, i.e., the etiology of renal and liver disease in GSD-Ia remains unclear, phenotypic heterogeneity and the lack of a stringent genotype-phenotype in GSD-Ia. It is necessary to conduct extensive genetic studies in the local population due to a high suspected incidence of disease, a lack of molecular genetic data, the clinical heterogeneity of GSD with challenging disease diagnosis,the high mortality and economic burden of end-stage disease treatment, such as liver transplantation. Early genetic diagnosis of affected individuals and their asymptomatic family members will be aided by these tests, which will enable regular follow-ups to enhance patient management and genetic counselling. Research for genotype-phenotype correlation of local GSD Ia patients is required to help our health care providers in better understanding of GSD’s clinical presentation, minimizing the risk of morbidity and mortality due to late diagnosis in the future. Common laboratory findings of GSD Ia and Ib are hypoglycemia, hyperlipidemia, hyperuricemia, and lactic acidemia. GSD type Ib, shows systemic infections like stomatitis, Crohn-like enteritis because of neutropenia, neutrophil, and monocyte dysfunction [21], these symptoms were not observed in enrolled cases in this study. 2-step diagnostic procedure without liver biopsy to confirm the clinical diagnosis of GSD Ia: the plasma biotinidase assay followed by the molecular analysis of the G6Pase gene are needed to avoid the misdiagnosis. Although inflammatory bowel disease is a characteristic of GSD 1b, but based on other symptoms and based on the diagnosis by the expert physicians *G6PC* gene was screened for variants in selected patients. Patients might be misdiagnosed due to phenotypic overlap, so screening of *SLC3A4* gene in the cases is our future consideration which was not possible now due to limited funding. There have been some studies on the clinical features and complications of GSD Ia in Pakistan, there is still a need for more research on the impact of the disease on patients’ quality of life. This could help inform more patient-centered approaches to care and improve outcomes for individuals with GSD Ia in Pakistan. Overall, there is a need for more research on GSD Ia in Pakistan, particularly in terms of epidemiology, genetics, treatment, and patient outcomes. Addressing these research gaps could help improve our understanding of the disease and lead to cost effective prevention and management strategies.

## Supporting information

S1 Table Frequency of demographic and clinical variants in 40 enrolled cases. (DOCX)

S2 Table Unidentified individuals data. (DOCX)
