# Supplementary material for: Mutational analysis and clinical investigations of medically diagnosed GSD 1a patients from Pakistan
Source: PLoS One. 2023 Nov 30;18(11):e0288965. doi: 10.1371/journal.pone.0288965 (PMC10688888; doi:10.1371/journal.pone.0288965)
Supplement: S1 Table — (DOCX) [file pone.0288965.s001.docx]

**Supplementary table 1: Frequency of demographic and clinical variants in 40 enrolled cases**

| **Clinicals** | **Categories** | **Frequency (%)** |
| --- | --- | --- |
| Gender | F | 14 (35) |
|  | M | 26 (65) |
| PCM | N | 5 (12.5) |
|  | Y | 35 (87.5) |
| Age | 0-5 | 14 (35) |
|  | 6-10 | 14 (35) |
|  | 11-15 | 10 (25) |
|  | 16-20 | 1 (2.5) |
|  | 21-25 | 1 (2.5) |
| Family history | N | 17 (42.5) |
|  | Y | 23 (57.5) |
| Status | Alive | 17 (42.5) |
|  | Decreased | 23 (57.5) |
| Short stature | N | 28 (70) |
|  | Y | 12 (30) |
| Hepatomegaly | N | 0 (0) |
|  | Y | 40 (100) |
| Anemia | N | 8 (20) |
|  | Y | 32 (80) |
| Hepaticadenomas | N | 38 (95) |
|  | Y | 2 (5) |
| Seizures | N | 30 (75) |
|  | Y | 10 (25) |
| Epistaxis | N | 33 (82.5) |
|  | Y | 7 (17.5) |
| Delayed motor development | N | 35 (87.5) |
|  | Y | 5 (12.5) |
| Cushingoid appearance | N | 37 (92.5) |
|  | Y | 3 (7.5) |
| Osteopenia | N | 31 (77.5) |
|  | Y | 9 (22.5) |
| Inflammatory bowel disease | N | 25 (62.5) |
|  | Y | 15 (37.5) |
