# Supplementary material for: Mutational analysis and clinical investigations of medically diagnosed GSD 1a patients from Pakistan
Source: PLoS One. 2023 Nov 30;18(11):e0288965. doi: 10.1371/journal.pone.0288965 (PMC10688888; doi:10.1371/journal.pone.0288965)
Supplement: S2 Table — (DOCX) [file pone.0288965.s002.docx]

**Supplementary table 2: Unidentified individuals data**

| **P- ID** | **PCM** | **Fam H** | **Hypoglycemia mg/dL** | **Hepatomegaly** | **Anemia** | **High microalbuminuria** | **Hepatic adenomas** | **Lactic Acidosis** | **Seizures** | **Epistaxis** | **delayed motor development** | **Cushingoid appearance** | **Osteopenia** | **Inflammatory bowel disease** |
| --- | --- | --- | --- | --- | --- | --- | --- | --- | --- | --- | --- | --- | --- | --- |
| GSD 1 | Y | N | 54 | Y | N | 35 | N | 6.8 | Y | N | Y | N | N | N |
| GSD 3 | Y | Y | 43 | Y | Y | 75 | N | 4.9 | N | N | N | N | Y | N |
| GSD 6 | Y | Y | 66 | Y | Y | 210 | N | 7 | N | N | N | N | Y | N |
| GSD 7 | Y | N | 59 | Y | Y | 107 | N | 6.8 | N | N | N | N | N | Y |
| GSD 8 | Y | N | 52 | Y | Y | 310 | N | 6.5 | Y | N | N | N | N | N |
| GSD 10 | Y | N | 56 | Y | Y | 115 | N | 5.8 | N | N | N | N | Y | Y |
| GSD 12 | Y | N | 56 | Y | Y | 350 | N | 6.5 | N | N | N | N | N | N |
| GSD 13 | N | N | 69 | Y | Y | 250 | N | 6.4 | Y | N | N | N | N | Y |
| GSD 14 | N | N | 65 | Y | Y | 230 | N | 5.8 | N | Y | N | N | N | N |
| GSD 15 | N | N | 66 | Y | Y | 301 | N | 5.5 | N | N | Y | N | N | N |
| GSD 16 | N | N | 46 | Y | N | 220 | N | 6.2 | N | N | N | N | Y | Y |
| GSD 17 | Y | N | 56 | Y | Y | 145 | N | 6.4 | N | N | N | N | N | N |
| GSD 18 | Y | N | 59 | Y | Y | 150 | N | 4.5 | Y | N | N | N | N | Y |
| GSD 20 | Y | Y | 48 | Y | Y | 220 | N | 6 | N | N | N | Y | N | N |
| GSD 21 | Y | Y | 66 | Y | N | 85 | N | 5 | N | N | N | N | N | Y |
| GSD 22 | Y | Y | 59 | Y | Y | 75 | N | 6.9 | N | N | N | N | Y | N |
| GSD 23 | Y | Y | 52 | Y | Y | 114 | N | 5.5 | N | N | Y | N | N | Y |
| GSD 24 | Y | Y | 58 | Y | Y | 45 | N | 6.4 | Y | N | N | N | N | N |
| GSD 25 | N | Y | 56 | Y | Y | 315 | N | 4.5 | N | N | N | N | N | N |
| GSD 26 | Y | Y | 61 | Y | Y | 45 | N | 5.5 | N | Y | N | N | N | Y |
| GSD 27 | Y | Y | 56 | Y | Y | 50 | N | 6 | N | N | N | N | N | N |
| GSD 28 | Y | Y | 69 | Y | Y | 145 | N | 5 | Y | N | N | N | N | N |
| GSD 29 | Y | Y | 65 | Y | Y | 305 | N | 6.4 | N | N | N | N | Y | Y |
| GSD 30 | Y | Y | 66 | Y | N | 112 | N | 4.5 | N | N | N | N | N | N |
| GSD 31 | Y | Y | 48 | Y | Y | 212 | N | 5.5 | N | N | Y | N | N | N |
| GSD 32 | Y | Y | 66 | Y | N | 350 | N | 6 | Y | Y | N | N | N | Y |
| GSD 33 | Y | Y | 59 | Y | Y | 100 | N | 5 | N | N | N | N | Y | N |
| GSD 34 | Y | Y | 52 | Y | Y | 89 | N | 4.5 | N | N | N | N | N | N |
| GSD 35 | Y | Y | 58 | Y | N | 65 | N | 5.5 | N | N | N | N | N | Y |
| GSD 36 | Y | N | 56 | Y | N | 400 | Y | 6 | Y | N | N | N | Y | Y |
| GSD 37 | Y | Y | 61 | Y | Y | 56 | N | 5 | N | N | N | N | N | N |
| GSD 38 | Y | Y | 56 | Y | N | 50 | N | 6.4 | Y | N | N | N | Y | N |
| GSD 39 | Y | Y | 69 | Y | Y | 305 | N | 4.5 | N | N | N | N | N | Y |
| GSD 40 | Y | N | 65 | Y | Y | 110 | N | 4 | N | N | N | N | N | Y |
